# Disease Awareness in Patients With Type 2 Diabetes: Analysis of Baseline Data From the SMART-Finder Observational Study

**DOI:** 10.2196/60246

**Published:** 2025-02-18

**Authors:** Christian Mueller, Thomas Neusser, Inga Thate-Waschke, Julia Nowicki, Tomasz Plominski, Regine Griesinger, Stefanie Kessner, Stephan Martin

**Affiliations:** 1 Pharmaceuticals Medizin, Pharmaceuticals, BAYER Vital GmbH, K56, Leverkusen, 51368, Germany, 49 1753005134; 2 smartpatient GmbH, Munich, Germany; 3 Emmes Biopharma Germany GmbH, Munich, Germany; 4 Verbund Katholischer Kliniken Duesseldorf (VKKD) und Westdeutsches Diabetes- und Gesundheitszentrum (WDGZ), Duesseldorf, Germany

**Keywords:** app-based documentation, chronic kidney disease, CKD, disease awareness, MyTherapy, type 2 diabetes, type 2 diabetes mellitus, urine albumin-to-creatinine ratio screening, patient, observational study, Germany, quality of life, treatment, therapy, physician-patient communication

## Abstract

**Background:**

Chronic kidney disease (CKD) is a common comorbidity of type 2 diabetes mellitus (T2DM). Data on the determination of CKD-related biomarkers among patients with T2DM in a real-life setting within Germany are limited.

**Objective:**

We aimed to determine the prevalence of CKD and risk factors, availability of urine albumin-to-creatinine ratio (UACR) and estimated glomerular filtration rate (eGFR) values, treatment satisfaction, and quality of life among patients with T2DM in Germany.

**Methods:**

SMART-Finder is a retrospective and prospective, observational, digital, patient-centered cohort study being performed as part of the routine use of an adherence-supporting app. This baseline analysis’ observation period was from August to November 2023. Patients with T2DM in Germany who actively used the MyTherapy app; allowed push notifications; and documented use of diabetes medications, renin-angiotensin system inhibitors, finerenone, and/or blood glucose test strips were eligible for inclusion. Study materials (background information, electronic consent form, and laboratory and electronic questionnaires) were provided to eligible patients via app push notifications. Participants completed an electronic case report form that included questions on their blood pressure; their most recent UACR, eGFR, and glycated hemoglobin (HbA_1c_) values in the past 12 months; the EQ-5D-5L; and the Diabetes Treatment Satisfaction Questionnaire. The primary outcome was the proportion of patients with a UACR of ≥30 mg/g.

**Results:**

Of 9527 invited eligible patients, 101 completed the electronic case report form (male: n=61; female: n=40; age: mean 54.2, SD 11.4 y). Of these, 1 female patient and 5 male patients reported their UACR values; 3 (all male) had a UACR of ≥30 mg/g. The remaining 95 patients reported that their health care professionals had not provided UACR measurements. Only 9 (8.9%) patients were aware of their latest eGFR values (3 patients: 15‐44 mL/min/1.73 m^2^; 6 patients: 45‐89 mL/min/1.73 m^2^), 90 provided HbA_1c_ values (80 patients: ≥6.0%), 46 had a systolic blood pressure of ≥130 mm Hg, and 83 reported former or current nephrotoxic medication intake. The mean EQ-5D-5L index score was 0.7 (SD 0.3; range –0.1 to 1.0; 50 patients). The mean Diabetes Treatment Satisfaction Questionnaire score was 28.8 (SD 6.8; range 9.0-36.0; 49 patients).

**Conclusions:**

Patients with T2DM who were using an adherence-supporting app in Germany lacked awareness of CKD-related biomarkers but had high knowledge of self-manageable biomarkers (eg, blood pressure, serum fasting glucose, and HbA_1c_ values). Our results suggest that treating physicians either do not test for UACRs and eGFRs or do not inform patients about the results. Nonadherence to diagnostic testing guidelines and a lack of physician-patient communication put patients at risk. Another reason for this health literacy imbalance may be the focus on HbA_1c_ instead of kidney comorbidity in patient education material. Future goals for diabetes management must include guideline-compliant testing of CKD-related biomarkers and open physician-patient communication.

## Introduction

Chronic kidney disease (CKD) is a common comorbidity of type 2 diabetes mellitus (T2DM), which is a leading cause of death and disability worldwide [1-8]. Patients with CKD show persistently decreased estimated glomerular filtration rates (eGFRs) or persistently elevated urine albumin excretion [9-11], which may progress to end-stage renal disease [12]. A high-normal urine albumin-to-creatinine ratio (UACR) is associated with a significantly increased risk of all-cause mortality [13].

Effective therapeutic approaches that can influence the development of CKD in patients with T2DM are available [14-16]. The current consensus guideline of the American Diabetes Association (ADA) and the Kidney Disease: Improving Global Outcomes (KDIGO) Work Group recommends renin-angiotensin system inhibitors, sodium-glucose cotransporter 2 inhibitors (SGLT2is), statins, and metformin as first-line drug therapies for patients with T2DM and CKD [17]. Glucagon-like peptide-1 receptor agonists are recommended if SGLT2is and metformin do not achieve sufficient glycemic control. For patients with persistent albuminuria despite standard treatment with glucose-lowering and antihypertensive medications, a nonsteroidal mineralocorticoid receptor antagonist is recommended in addition to baseline therapy [17].

The early identification of patients with diabetes who are at increased risk for renal disease is highly recommended [18,19]. Therefore, the eGFR (based on serum creatinine) and albuminuria (based on the UACR) should be assessed once per year in patients with T2DM [20,21]. As the UACR demonstrates a high degree of within-individual variability among individuals, regularly collecting samples for UACR testing may improve capacity for monitoring changes over time in clinical and research settings [22]. However, data on the determination of UACRs in a real-life setting and the prevalence of CKD among patients with T2DM in Germany are limited [23-26].

The SMART-Finder study was designed to determine the prevalence of CKD and risk factors, availability of UACR and eGFR values, satisfaction with treatment, and quality of life among patients with T2DM using an adherence-supporting app in Germany [27]. In this analysis of baseline data from the SMART-Finder study, the primary objective was to assess the proportion of patients with T2DM who had an elevated UACR (≥30 mg/g; albumin-to-creatinine ratio [ACR] stages A2 and A3). As secondary objectives, the proportion of patients without UACR test data was determined, blood pressure and nephrotoxic comedication status were assessed, and quality of life was analyzed. Disease awareness and treatment satisfaction were assessed as further secondary objectives.

## Methods

### Study Design

As previously described [27], the recruitment of patients and documentation of data for this retrospective and prospective, observational, digital, patient-centered cohort study were carried out without the assistance of a health care professional (HCP), as part of the routine use of the MyTherapy app (smartpatient GmbH) by patients with T2DM in Germany. The app helps patients manage their treatment-related tasks and access information on other health topics, including comorbidities, by using their mobile device. The app provides the user with a daily “To do” list of tasks, including those regarding medication intake, and asks the user to confirm whether each task has been completed [27]. Patients who documented the following medications or medical devices within the app were considered eligible for participation during initial selection: metformin, acarbose, dipeptidyl-peptidase-4 inhibitor, glucagon-like peptide-1 receptor agonist, renin-angiotensin system inhibitor, SGLT2i, basal insulin, nonsteroidal mineralocorticoid receptor antagonist, sulfonylurea, and blood glucose test strips.

To ensure that patients were properly informed, all eligible patients who actively used the app and allowed push notifications during the app’s regular operation received content via push notifications during an awareness phase, which lasted from August 2 to 15, 2023. This content comprised 3 videos about the scope and goals of the SMART-Finder study, the risk of CKD in patients with T2DM, and the impairment of kidney function in patients with CKD; a study description page; a document for collecting laboratory values; and an electronic consent form for explaining, in detail, which data were used for this study and obtaining consent for the use of pseudonymized data. After completion of the consent form, an electronic case report form (eCRF), the EQ-5D-5L, and the Diabetes Treatment Satisfaction Questionnaire (DTSQ) were provided in the “Discover” section of the app (patients were notified about this via push notifications as well) on August 18, 21, and 24, 2023, respectively. The eCRF included questions on patients’ residential region, age, gender (self-reported as 1 of 3 options: male, female, or diverse), weight, height, smoking status, last systolic and diastolic blood pressures, serum fasting glucose level, and most recent available laboratory values within the last 12 months (UACR, eGFR, and glycated hemoglobin [HbA_1c_]). If patients did not know these laboratory values, they were encouraged to request them from their treating physician.

The observation period for the baseline analysis was from August 18, 2023, until the cutoff date (November 20, 2023). Patients who signed the informed consent form and completed at least the eCRF were eligible for baseline analysis.

### Ethical Considerations

Ethics approval for this study was granted by the Ethics Committee of the North Rhine Medical Association (Ärztekammer Nordrhein) on October 13, 2022 (approval number: 2022263). All patients were offered the informed consent form; of the 103 who signed the form, 2 withdrew consent. All patient data were handled in a pseudonymized manner, and the privacy of patients was ensured. Upon completion of the baseline eCRF (regardless of EQ-5D-5L or DTSQ completion), patients received a €10 (US $10.80) voucher for Shop Apotheke as compensation for their time spent on data documentation.

### Statistical Analysis

Data were analyzed descriptively with SAS (release 9.4 or higher; SAS Institute Inc). UACRs were categorized based on KDIGO ACR stages [6], with the addition of a fourth level indicating nephrotic-range albuminuria [28,29] (stage A1: UACR of <30 mg/g; stage A2: UACR of 30‐300 mg/g; stage A3: UACR of 301‐3000 mg/g; stage A4: UACR of >3000 mg/g). Serum fasting glucose levels were categorized based on the ADA diagnostic criteria for diabetes and prediabetes [30], and eGFRs were reported by KDIGO category [6]. The EQ-5D-5L index score was calculated by using a value set for the German general population [31].

## Results

### Study Population

**Figure 1.**
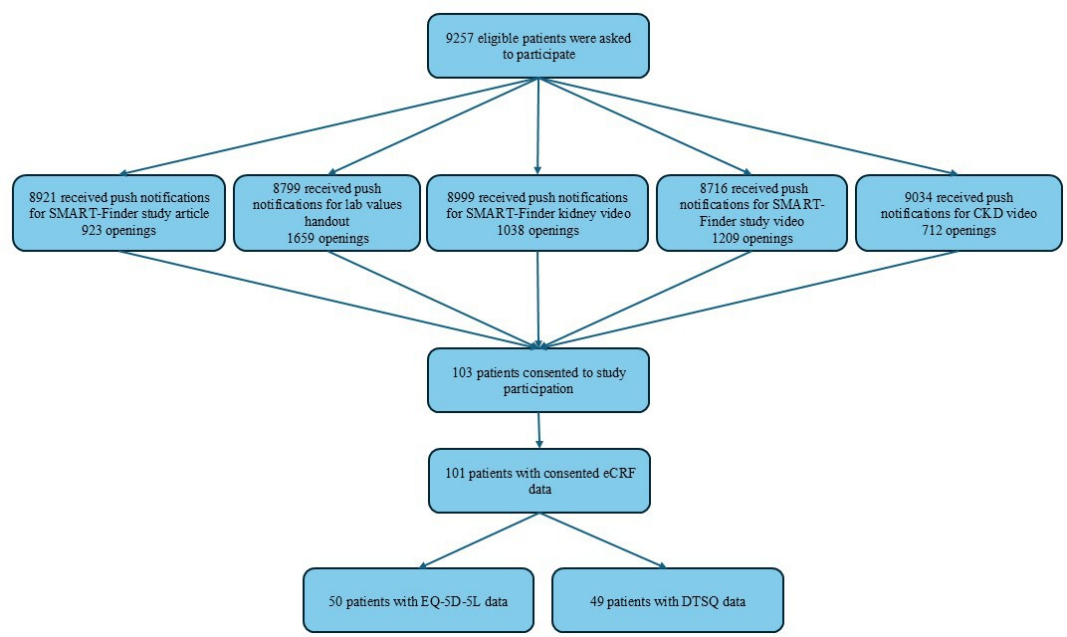
Patient recruitment and eligibility in the SMART-Finder observational study. Push notifications were sent out to all eligible patients with T2DM (N=9527). The number of patients receiving each push notification could be influenced by factors such as internet connectivity or patients having their devices switched off at the time of the notification. Therefore, 8921 (93.6%), 8799 (92.4%), 8999 (94.5%), 8716 (91.5%), and 9034 (94.8%) of the 9527 eligible patients received push notifications for the article on the SMART-Finder study, the laboratory values handout, the video on kidney function in CKD, the video on the SMART-Finder study, and the video on risk of CKD, respectively. CKD: chronic kidney disease; DTSQ: Diabetes Treatment Satisfaction Questionnaire; eCRF: electronic case report form; T2DM: type 2 diabetes mellitus.

During the awareness campaign, push notifications for study- and indication-related content were sent out to a cohort of patients with T2DM (Figure 1), whereof 8799 to 9527 patients received these push notifications. Of these 8799 to 9527 patients, 103 provided consent, and 2 withdrew consent. A total of 101 patients completed the eCRF and were therefore included in the baseline analysis.

Of the 101 participating patients with T2DM, 61 were male, and 40 were female. The study population had a mean age of 54.2 (SD 11.4; range 22‐76) years, a mean weight of 103.9 (SD 21.7; range 45‐149) kg, and a mean BMI of 34 (SD 7.0; range 16.9‐54.7) kg/m^2^. Further, 26 patients were smokers, 43 were former smokers, and 32 had never smoked.

### Laboratory Values and Blood Pressure

Of the 101 patients in the analyzed cohort, only 1 female patient and 5 male patients reported their UACR values (Table 1); 3 of the patients (all male) had a UACR of ≥30 mg/g. The remaining 95 (94.1%) patients reported that their HCPs had not provided UACR measurements. For eGFR values, a similar finding was observed—only 9 (8.9%) of the 101 patients were aware of their latest values.

**Table 1.** Classification of UACRs^a^ in female and male patients.

	Total patients (N=101), n (%^b^)	Patients stratified by UACR, n (%^b^)				
		Missing data (n=95)	<30 mg/g (n=3)	30‐300 mg/g (n=2)	301‐3000 mg/g (n=1)	>3000 mg/g (n=0)
Female	40 (39.6)	39 (41)	1 (33)	0 (0)	0 (0)	0 (0)
Male	61 (60.4)	56 (59)	2 (67)	2 (100)	1 (100)	0 (0)

a UACR: urinary albumin-to-creatinine ratio.

b Percentages were calculated by using the n values in the column headings as the denominator.

In contrast to laboratory values provided by HCPs, self-determined test results were more commonly reported. In total, 65 (64.4%) patients provided their serum fasting glucose levels, and 90 (89.1%) patients provided their latest HbA_1c_ values (Table 2).

**Table 2.** eGFR^a^, HbA_1c_^b^^,^^c^, and serum fasting glucose^d^ at baseline.

	Patients (N=101), n (%)
eGFR at baseline, mL/min/1.73m^2^	
Missing	92 (91.1)
15‐29	1 (1)
30‐44	2 (2)
45‐59	2 (2)
60‐89	4 (4)
HbA_1c_ at baseline, %	
Missing	11 (10.9)
<6.0	9 (8.9)
6.0‐6.5	29 (28.7)
6.6‐7.0	16 (15.8)
7.1‐7.5	16 (15.8)
7.6‐8.0	6 (5.9)
8.1‐8.5	3 (3)
8.6‐9.0	8 (7.9)
>9.0	3 (3)
Serum fasting glucose summary at baseline, mg/dL	
Missing	36 (35.6)
<100	7 (6.9)
100‐126	38 (37.6)
>126	20 (19.8)

a eGFR: estimated glomerular filtration rate.

b HbA_1c_: glycated hemoglobin.

c To convert HbA_1c_ to mmol/mol, multiply by 10.93 and subtract 23.5 mmol/mol.

d To convert serum fasting glucose to mmol/L, divide by 18.018.

Systolic and diastolic blood pressures are presented in Table 3. In total, 31 (30.7%) patients had a high-normal systolic blood pressure, 15 (14.9%) patients showed systolic hypertension, and 13 (12.9%) patients showed diastolic hypertension according to the 2023 European Society of Hypertension guidelines for the management of arterial hypertension (assuming office-based blood pressure measurements) [32].

**Table 3.** Systolic and diastolic blood pressures at baseline.

	Patients (N=101), n (%)
Systolic blood pressure at baseline, mm Hg	
≤119	18 (17.8)
120‐129	37 (36.6)
130‐139	31 (30.7)
140‐159	13 (12.9)
160‐179	2 (2)
Diastolic blood pressure at baseline, mm Hg	
55‐59	4 (4)
60‐64	4 (4)
65‐69	7 (6.9)
70‐74	14 (13.9)
75‐79	11 (10.9)
80‐84	36 (35.6)
85‐89	12 (11.9)
90‐99	9 (8.9)
100‐109	4 (4)

### Medication Usage

Use of SGLT2is, the nonsteroidal mineralocorticoid receptor antagonist finerenone, hypertension medication (defined by anatomic therapeutic chemical codes), and nephrotoxic medication (as listed by Patel and Sapra [33]) was reported by 44, 0, 80, and 83 patients, respectively. All patients confirmed their T2DM diagnosis at baseline. Further, 81 reported hypertension (77 with treated hypertension and 4 with untreated hypertension) and 12 reported CKD as comorbidities. Reported comedication data for all patients are presented in Multimedia Appendix 1.

### Quality of Life and Treatment Satisfaction

A total of 50 patients completed the EQ-5D-5L. The mean EQ-visual analog scale score was 64.4 (SD 24.4; range 3.0‐99.0; on a scale of 0‐100, where 0 is the worst and 100 is the best health state possible), and the mean EQ-5D-5L index score was 0.7 (SD 0.3; range –0.1 to 1.0). The DTSQ was completed by 49 patients, with a mean DTSQ score of 28.8 (SD 6.8; range 9.0‐36.0) at baseline.

## Discussion

### Principal Results and Comparison With Prior Work

With the SMART-Finder study, we are investigating the self-reporting of UACR, eGFR, and HbA_1c_ values in a cohort of patients with T2DM using an adherence-supporting app in Germany. This baseline analysis provides insights into the implementation of treatment guidelines and HCP-patient interactions.

We observed a low participation rate in our cohort of patients, who were taking responsibility for their health status by regularly tracking adherence. Of the 9527 patients who were contacted, only 712 (7.5%) to 1659 (17.4%) patients engaged with at least one aspect of the awareness campaign by clicking on a video or the written information content in the app. The final participation rate—8.4% (101 of 1209 patients who saw the SMART-Finder study video)—is comparable to those observed in other awareness campaigns; for example, 10.2% (804/7865) and 10.1% (802/7920) of invited participants completed a survey on a public health campaign for increasing kidney health awareness in a Canadian province [34].

In contrast to activities that can be self-managed by patients (blood pressure measurement and testing of HbA_1c_ and serum fasting glucose levels), about which our cohort appears to be well informed, our results suggest a dramatic lack of awareness of renal function status in this population at risk of CKD. This is of particular concern, since almost half of the cohort (46/101, 45.5%) had a systolic blood pressure of ≥130 mm Hg, and 82.2% (83/101) reported former or current intake of nephrotoxic medication. Further, more than 90% of the patients were unaware of their eGFR (92/101, 91.1%) and UACR (95/101, 94.1%), but 89.1% (90/101) of the patients knew their HbA_1c_ level, suggesting that treating physicians either do not test for UACRs and eGFRs or do not inform patients about the results. Both a lack of adherence to guidelines for diagnostic testing and a lack of HCP-patient communication put patients at risk. Another reason for this imbalance in health literacy may be the focus on HbA_1c_, rather than on kidney comorbidity, in patient education material.

### Limitations

The presented baseline analysis has some limitations. There is a selection bias, as only users of the adherence-supporting app, that is, those who seemed to have experience with using technology, were able to participate. Additionally, the unconventional method of data collection, with patients requesting data from HCPs, may have introduced reporting bias from the HCPs. Further, due to the low participation rate and the lack of UACR and eGFR values, our findings on CKD risk in the observed cohort of patients may not be generalizable to all patients with T2DM. However, the missing data show a lack of awareness in a high-risk population. Even though the annual eGFR and albuminuria testing rates in Germany are 96.5% and 77.2%, respectively, our results suggest that among German patients with diabetes, knowledge regarding their kidney function and disease is still insufficient [35].

### Conclusions

Baseline data from the SMART-Finder study, which were derived from patients with T2DM who were using an adherence-supporting app in Germany, showed a dramatic lack of awareness of kidney-related biomarkers (UACR and eGFR) but revealed high knowledge of HbA_1c_ values. As only informed patients are empowered patients, future goals for diabetes management must include guideline-compliant testing of CKD-related biomarkers and open communication between HCPs and patients.

## Supplementary material

10.2196/60246 Multimedia Appendix 1 Reported concomitant medications.

## Abbreviations

ACR: albumin-to-creatinine ratio
ADA: American Diabetes Association
CKD: chronic kidney disease
DTSQ: Diabetes Treatment Satisfaction Questionnaire
eCRF: electronic case report form
eGFR: estimated glomerular filtration rate
HbA_1c_: glycated hemoglobin
HCP: health care professional
KDIGO: Kidney Disease: Improving Global Outcomes
SGLT2i: sodium-glucose cotransporter 2 inhibitor
T2DM: type 2 diabetes mellitus
UACR: urine albumin-to-creatinine ratio
