# Supplementary material for: Disease Awareness in Patients With Type 2 Diabetes: Analysis of Baseline Data From the SMART-Finder Observational Study
Source: JMIR Form Res. 2025 Feb 18;9:e60246. doi: 10.2196/60246 (PMC11853924; doi:10.2196/60246)
Supplement: Multimedia Appendix 1 [file formative-v9-e60246-s001.docx]

## Multimedia Appendix 1

**Table.** Reported concomitant medications.^a^

| **Concomitant medication** | **Patients, n (%) (N = 101)** | |
| --- | --- | --- |
| ANY | 100 | (99.0) |
| 50 + KAPSELN | 1 | (1.0) |
| ABASAGLAR | 1 | (1.0) |
| ACETOLYT | 1 | (1.0) |
| ADENURIC | 1 | (1.0) |
| AGOMELATIN | 1 | (1.0) |
| AKKU CHEK FASTCLIX | 1 | (1.0) |
| AKKU TEST | 1 | (1.0) |
| ALDACTONE | 2 | (2.0) |
| ALFUZOSIN | 1 | (1.0) |
| ALLE ABENDMEDIS | 1 | (1.0) |
| ALLOPURINOL | 16 | (15.8) |
| ALPHA-LIPONSAURE | 1 | (1.0) |
| AMINEURIN | 1 | (1.0) |
| AMITRIPTYLIN | 1 | (1.0) |
| AMLODIPIN | 18 | (17.8) |
| AMLODIPIN (BESILAT) | 2 | (2.0) |
| AMLODIPIN BESILAT | 1 | (1.0) |
| AMLODIPIN/VALSARTAN/HCT BETA | 1 | (1.0) |
| AMOROLFIN | 1 | (1.0) |
| ANORO | 1 | (1.0) |
| ANTIBIOTIKA | 1 | (1.0) |
| ANTILIA | 1 | (1.0) |
| APIDRA | 1 | (1.0) |
| ARNIKASALBE | 1 | (1.0) |
| ASS | 16 | (15.8) |
| ATACAND PROTECT | 1 | (1.0) |
| ATMADISC | 1 | (1.0) |
| ATORVASTATIN | 25 | (24.8) |
| ATROVASTATIN | 1 | (1.0) |
| B12 | 2 | (2.0) |
| BALDRIAN | 1 | (1.0) |
| BD MICROFINE | 1 | (1.0) |
| BETAVERT | 1 | (1.0) |
| BETMIGA | 1 | (1.0) |
| BICANORM | 2 | (2.0) |
| BIMATOPROST | 1 | (1.0) |
| BIO BOCKSHORNKLEE + PIOERIN | 1 | (1.0) |
| BIOFANAL | 1 | (1.0) |
| BION3 ENERGY | 1 | (1.0) |
| BIOTIN | 1 | (1.0) |
| BIRAMLO | 1 | (1.0) |
| BISO LICH | 3 | (3.0) |
| BISOHEXAL | 6 | (5.9) |
| BISOLICH | 1 | (1.0) |
| BISOPROLOL | 10 | (9.9) |
| BRIMO-VISION SINE | 1 | (1.0) |
| BRINZOLAMID | 1 | (1.0) |
| BUPROPION | 1 | (1.0) |
| CALCIGEN D CITRO | 1 | (1.0) |
| CALCIPOTRIOL | 1 | (1.0) |
| CALCIUM | 1 | (1.0) |
| CANDAXIRO | 3 | (3.0) |
| CANDEAMLO | 1 | (1.0) |
| CANDECOR | 1 | (1.0) |
| CANDECOR COMP | 1 | (1.0) |
| CANDESARAN | 1 | (1.0) |
| CANDESARTAN | 19 | (18.8) |
| CAPROS | 1 | (1.0) |
| CAROTIN | 1 | (1.0) |
| CARVE TAD | 1 | (1.0) |
| CARVEDILOL | 1 | (1.0) |
| CATAPRESAN | 1 | (1.0) |
| CELECOXIB | 1 | (1.0) |
| CETIRIZIN | 3 | (3.0) |
| CHLORPROTHIXEN | 1 | (1.0) |
| CHLORTALIDON | 1 | (1.0) |
| CITALOPRAM | 3 | (3.0) |
| CLOPIDOGREL | 1 | (1.0) |
| COLECALCIFEROL | 3 | (3.0) |
| COSENTYX | 1 | (1.0) |
| CRANBERRY | 1 | (1.0) |
| DEKRISTOL | 12 | (11.9) |
| DESOFENANCE | 1 | (1.0) |
| DESOGESTREL | 3 | (3.0) |
| DEXAMETHASON | 1 | (1.0) |
| DIAZEPAM | 1 | (1.0) |
| DICLOFENAC | 1 | (1.0) |
| DICLOFENAC-NATRIUM | 1 | (1.0) |
| DOPPELHERZ BEI REIZDARM | 1 | (1.0) |
| DOPPELHERZ FESTE NAGEL EXTRA | 1 | (1.0) |
| DORZOCOMP-VISION | 1 | (1.0) |
| DOXAGAMMA | 1 | (1.0) |
| DOXAZOSIN | 1 | (1.0) |
| DOXEPIN | 1 | (1.0) |
| DULOXETIN | 2 | (2.0) |
| ELIQUIS | 1 | (1.0) |
| ELITYRAN | 1 | (1.0) |
| ELONTRIL | 1 | (1.0) |
| ENOXAPARIN BECAT | 1 | (1.0) |
| EPLERENON | 1 | (1.0) |
| ERGENYL CHRONO | 1 | (1.0) |
| ERLEADA | 1 | (1.0) |
| ESCITALOPRAM | 2 | (2.0) |
| ESOMEPRAZOL | 2 | (2.0) |
| ESTRADIOL | 1 | (1.0) |
| ETORICOXIB | 1 | (1.0) |
| EUTHYROX | 4 | (4.0) |
| EZETIMIB | 6 | (5.9) |
| EZETIMIB/SIMVASTATIN | 2 | (2.0) |
| FAMENITA | 1 | (1.0) |
| FENOFIBRAT | 1 | (1.0) |
| FERRO OPTIMUM | 1 | (1.0) |
| FERRO SANOL DUODENAL | 2 | (2.0) |
| FIASP | 1 | (1.0) |
| FLUOXETIN | 1 | (1.0) |
| FLUTIFORM | 1 | (1.0) |
| FOLSAN | 1 | (1.0) |
| FOLSAURE | 6 | (5.9) |
| FORXIGA | 17 | (16.8) |
| FOSTER | 3 | (3.0) |
| FUROSEMID | 1 | (1.0) |
| GABAPENTIN | 2 | (2.0) |
| GAVISCON DUAL | 1 | (1.0) |
| GINGOBETA | 1 | (1.0) |
| GINKOBIL | 1 | (1.0) |
| GLIMEPIRID | 2 | (2.0) |
| GRANUFINK FEMINA | 1 | (1.0) |
| GYNOKADIN | 1 | (1.0) |
| HCT | 2 | (2.0) |
| HERZASS | 1 | (1.0) |
| HYDROCORTISON | 1 | (1.0) |
| HYDROMORPHON | 1 | (1.0) |
| HYGROTON | 4 | (4.0) |
| IBU | 2 | (2.0) |
| IBUFLAM | 1 | (1.0) |
| INDAPAMID | 4 | (4.0) |
| INFECTOGENTA | 1 | (1.0) |
| IRBESARTAN | 2 | (2.0) |
| IS 5MONO | 1 | (1.0) |
| ISICOM | 1 | (1.0) |
| IVABALAN | 1 | (1.0) |
| IVABRADIN | 1 | (1.0) |
| JANUMET | 3 | (3.0) |
| JANUVIA | 5 | (5.0) |
| JARDIANCE | 26 | (25.7) |
| JOHANNISKRAUT | 2 | (2.0) |
| JUFORMIN | 3 | (3.0) |
| KALINOR | 1 | (1.0) |
| KALINOR RETARD P | 1 | (1.0) |
| KIJIMEA K53 | 1 | (1.0) |
| KURKUMA + INGWER + WEIHRAUCH | 1 | (1.0) |
| L THYROX | 3 | (3.0) |
| L THYROXIN | 2 | (2.0) |
| L-THYROX | 2 | (2.0) |
| L-THYROXIN | 3 | (3.0) |
| LAIF | 1 | (1.0) |
| LANTAREL FS | 1 | (1.0) |
| LANTUS | 4 | (4.0) |
| LATANOPROST | 1 | (1.0) |
| LEFLUNOMID | 1 | (1.0) |
| LERCANIDIPIN | 5 | (5.0) |
| LETROZOL | 1 | (1.0) |
| LEVETIRACETAM | 2 | (2.0) |
| LEVODOPA PLUS BENSERAZID | 1 | (1.0) |
| LEVODOPA/BENSERAZID | 1 | (1.0) |
| LIMPTAR | 1 | (1.0) |
| LIPROLOG | 1 | (1.0) |
| LISINOPRIL | 1 | (1.0) |
| LOPERAMID | 1 | (1.0) |
| LORA | 2 | (2.0) |
| LORANO | 1 | (1.0) |
| MACROGOL | 1 | (1.0) |
| MADOPAR | 1 | (1.0) |
| MAGNESIUM | 7 | (6.9) |
| MAGNESIUM UND VITAMIN E | 1 | (1.0) |
| MARIENDISTELOL | 1 | (1.0) |
| MAXIM | 1 | (1.0) |
| MEDIKINET ADULT | 1 | (1.0) |
| MERFORMI | 1 | (1.0) |
| MESALAZIN | 1 | (1.0) |
| METEX | 1 | (1.0) |
| METFORMIN | 44 | (43.6) |
| METHOTREXAT | 1 | (1.0) |
| METHYLCOBALAMIN B12 | 1 | (1.0) |
| METHYLPHENIDAT-HCL | 1 | (1.0) |
| METOBETA | 1 | (1.0) |
| METOHEXAL | 1 | (1.0) |
| METOHEXAL-SUCC | 5 | (5.0) |
| METOPROLOL | 5 | (5.0) |
| METOPROLOL SUCCINAT | 3 | (3.0) |
| METOPROLOLSUCCINAT | 3 | (3.0) |
| MILGAMMA | 1 | (1.0) |
| MIRTALICH | 1 | (1.0) |
| MIRTAZAPIN | 3 | (3.0) |
| MITTAGS-MEDIS NEHMEN | 1 | (1.0) |
| MOMECUTAN | 1 | (1.0) |
| MOMETAHEXAL | 1 | (1.0) |
| MOXONIDIN | 3 | (3.0) |
| MYDOCALM | 1 | (1.0) |
| MYFORTIC | 1 | (1.0) |
| MYOPRIDIN | 1 | (1.0) |
| NEBIVOLOL | 2 | (2.0) |
| NEUREXAN | 1 | (1.0) |
| NEVANAC | 1 | (1.0) |
| NILEMDO | 1 | (1.0) |
| NITRAZEPAM | 1 | (1.0) |
| NIZORAL CREME | 1 | (1.0) |
| NOVALGIN | 1 | (1.0) |
| NOVAMINSULFON | 1 | (1.0) |
| NOVOFINE | 1 | (1.0) |
| OLMESARTAN | 1 | (1.0) |
| OMEGA 3 | 2 | (2.0) |
| OMEGA-3 | 1 | (1.0) |
| OMEP | 1 | (1.0) |
| OMEPRAZOL | 3 | (3.0) |
| OPC MONO | 1 | (1.0) |
| OPIPRAMOL | 1 | (1.0) |
| OXYBUGAMMA | 1 | (1.0) |
| OZEMPIC | 17 | (16.8) |
| PALEXIA | 1 | (1.0) |
| PALEXIA RETARD | 1 | (1.0) |
| PANTOPRAZOL | 23 | (22.8) |
| PANTOZOL | 1 | (1.0) |
| PAROXETIN | 2 | (2.0) |
| PHENPROGAMMA | 1 | (1.0) |
| PRADAXA | 1 | (1.0) |
| PRAMIPEXOL | 1 | (1.0) |
| PREDNISOLON | 1 | (1.0) |
| PREGABALIN | 7 | (6.9) |
| PREGABIN | 1 | (1.0) |
| PROGRAF | 1 | (1.0) |
| PROMETHAZIN | 1 | (1.0) |
| PROPRA | 1 | (1.0) |
| PROTHYRID | 1 | (1.0) |
| QUETIAPIN | 5 | (5.0) |
| R-ALPHA-LIPONSAURE | 1 | (1.0) |
| RAMILICH | 12 | (11.9) |
| RAMILICH COMP | 1 | (1.0) |
| RAMIPRIL | 15 | (14.9) |
| RAMIPRIL ARISTO PLUS AMLODIPIN | 1 | (1.0) |
| RANEXA | 2 | (2.0) |
| REDCARE IMMUN FORTE | 1 | (1.0) |
| RESTEX | 2 | (2.0) |
| ROPINIROL | 1 | (1.0) |
| ROSU | 1 | (1.0) |
| ROSUVASTATIN | 6 | (5.9) |
| ROSUZET | 2 | (2.0) |
| SECULACT | 1 | (1.0) |
| SERTRALIN | 2 | (2.0) |
| SIMPONI | 1 | (1.0) |
| SIMVA | 8 | (7.9) |
| SIMVASTATIN | 2 | (2.0) |
| SIOFOR | 5 | (5.0) |
| SIOFOR XR | 2 | (2.0) |
| SITAGLIPTIN | 2 | (2.0) |
| SITAGLIPTIN METFORMIN | 2 | (2.0) |
| SLINDA | 1 | (1.0) |
| SORTIS | 1 | (1.0) |
| SPIRONOLACTON | 5 | (5.0) |
| STEGLATRO | 1 | (1.0) |
| STEIROFEMIN | 1 | (1.0) |
| STRATTERA | 1 | (1.0) |
| SULIQUA | 1 | (1.0) |
| TADALAFIL | 2 | (2.0) |
| TAMSUBLOCK | 1 | (1.0) |
| TAMSULOSIN | 4 | (4.0) |
| TAPENTADOL | 2 | (2.0) |
| TECFIDERA | 1 | (1.0) |
| TELMISARTAN | 2 | (2.0) |
| TESTAVAN | 1 | (1.0) |
| TESTOGEL | 2 | (2.0) |
| THYRONAJOD | 2 | (2.0) |
| TILIDIN | 2 | (2.0) |
| TILIDIN COMP. | 1 | (1.0) |
| TIZANIDIN | 1 | (1.0) |
| TORASEMID | 13 | (12.9) |
| TOUJEO | 3 | (3.0) |
| TRAMADOL | 1 | (1.0) |
| TRAZODON | 1 | (1.0) |
| TRELEGY ELLIPTA | 2 | (2.0) |
| TRESIBA | 5 | (5.0) |
| TRIMIPRAMIN | 1 | (1.0) |
| TRULICITY | 14 | (13.9) |
| TRYPTOPHAN | 1 | (1.0) |
| ULTIBRO | 2 | (2.0) |
| VAGISAN | 1 | (1.0) |
| VAGISAN BIOTIN-LACTO | 1 | (1.0) |
| VALSACOR | 2 | (2.0) |
| VALSARTAN | 3 | (3.0) |
| VELMETIA | 3 | (3.0) |
| VENLAFAXIN | 8 | (7.9) |
| VERAPAMIL | 1 | (1.0) |
| VIACORAM | 1 | (1.0) |
| VIACORIND | 1 | (1.0) |
| VIANI | 1 | (1.0) |
| VICTOZA | 3 | (3.0) |
| VIGANTOL | 1 | (1.0) |
| VIGANTOLETTEN | 1 | (1.0) |
| VIGANTOLVIT | 1 | (1.0) |
| VITAMIN B KOMPLEX | 1 | (1.0) |
| VITAMIN B12 | 4 | (4.0) |
| VITAMIN C | 1 | (1.0) |
| VITAMIN D | 2 | (2.0) |
| VITAMIN D3 | 5 | (5.0) |
| VITAMIN E | 1 | (1.0) |
| VITAMIN K2 | 1 | (1.0) |
| VITAMIN-B-KOMPLEX | 3 | (3.0) |
| VOLTAREN PLUS | 1 | (1.0) |
| WEIßDORN | 1 | (1.0) |
| WLS OPTIMUM | 1 | (1.0) |
| XARELTO | 7 | (6.9) |
| XELEVIA | 2 | (2.0) |
| XIGDUO | 7 | (6.9) |
| XIPAMID | 1 | (1.0) |
| ZAHNE REIN | 1 | (1.0) |
| ZINK + HISTIDIN | 1 | (1.0) |

^a^Multiple responses possible. Medication names are as reported by the patients.
